# Nasal microbiota dominated by *Moraxella* spp. is associated with respiratory health in the elderly population: a case control study

**DOI:** 10.1186/s12931-020-01443-8

**Published:** 2020-07-14

**Authors:** Ellen H. A. van den Munckhof, Harriet C. Hafkamp, Josephine de Kluijver, Ed J. Kuijper, Maurits N. C. de Koning, Wim G. V. Quint, Cornelis W. Knetsch

**Affiliations:** 1 DDL Diagnostic Laboratory, Visseringlaan 25, 2288 ER Rijswijk, The Netherlands; 2 Department of Otorhinolaryngology, Reinier de Graaf Hospital, Delft, The Netherlands; 3 Department of Pulmonology, Reinier de Graaf Hospital, Delft, The Netherlands; 4 Department of Medical Microbiology, Leiden University Medical Centre, Leiden, The Netherlands

**Keywords:** Elderly, Microbiota, Nasal passages, Oropharynx, Respiratory tract infection

## Abstract

**Background:**

The elderly (≥65 years) are one of the populations most at risk for respiratory tract infections (RTIs). The aim of this study was to determine whether nasal and/or oropharyngeal microbiota profiles are associated with age and RTIs.

**Methods:**

Nasal and oropharyngeal swabs of 152 controls and 152 patients with an RTI were included. The latter group consisted of 72 patients with an upper respiratory tract infection (URTI) and 80 with a lower respiratory tract infection (LRTI). Both nasal and oropharyngeal swabs were subjected to microbiota profiling using amplicon sequencing of the 16S rRNA gene. *Moraxella* species were determined using quantitative real-time PCR and culture.

**Results:**

Based on the microbiota profiles of the controls and the patients with an RTI, eight nasal and nine oropharyngeal microbiota clusters were defined. Nasal microbiota dominated by either *Moraxella catarrhalis* or *Moraxella nonliquefaciens* was significantly more prevalent in elderly compared to mid-aged adults in the control group (*p* = 0.002). Dominance by *M. catarrhalis/nonliquefaciens* was significantly less prevalent in elderly with an LRTI (*p* = 0.001) compared to controls with similar age.

**Conclusions:**

Nasal microbiota dominated by *M. catarrhalis/nonliquefaciens* is associated with respiratory health in the elderly population.

## Background

Respiratory tract infections (RTIs) remain one of the leading causes of morbidity and mortality worldwide [1, 2]. Whereas upper respiratory tract infections (URTIs) are very common but rarely life threatening, lower respiratory tract infections (LRTIs) are responsible for more severe illnesses, like pneumonia. The populations at risk are the very young (< 5 years) and the elderly (≥ 65 years).

During the first year of life, host and environmental factors, such as genetic predisposition, mode of delivery, infant feeding, exposure to antibiotics, vaccination and geographic location, affect the development of the airway microbiota [3, 4]. For the nose, this results gradually in a microbiota profile dominated by *Dolosigranulum*, *Corynebacterium*, *Haemophilus*, *Moraxella*, *Staphylococcus* and/or *Streptococcus* spp. [5]*.* In the first years of life, microbiota profiles dominated by *Dolosigranulum* and/or *Corynebacterium* spp. are more stable and are positively associated with lower rates of RTIs [6–9]. Less stable microbiota profiles characterized by the high abundance of the oral bacteria *Haemophilus* and *Streptococcus* spp. are associated with a higher likelihood of an RTI and their proportion is significantly higher in samples obtained during RTIs when compared to ‘healthy’ samples [6–10]. Furthermore, these microbiota profiles have been associated with an increased risk of recurrent wheeze and asthma in later childhood [9]. For microbiota profiles dominated by *Moraxella* spp. variable results have been reported regarding their stability and association with RTIs [6–9]. The differences in susceptibility to RTIs likely arise from a complex interplay between mucosa, innate and adaptive immunity, and airway microbiota.

In elderly, the mechanisms of the heightened susceptibility to RTIs are still poorly understood. Immunosenescence, defined as age-related deterioration of both innate and adaptive immunity, seems to impair elderly to elicit effective immune responses against pathogens [11]. In addition, immunosenescence might influence the composition of the human microbiota [12, 13]. Only few studies have addressed the upper airway microbiota in elderly [14–17], and even less in relation to RTIs [18]. The available study typically focusses on the oropharynx for LRTIs, which is suggested to be the main source of microorganisms to the lower airways in adults [19]. They observed three microbiota profiles strongly associated with pneumonia and either dominated by *Lactobacillus*, *Rothia* or *Streptococcus (pseudo)pneumoniae*. In contrast, three other microbiota clusters were correlated with respiratory health and were all characterized by more diverse profiles containing higher abundances of especially *Prevotella*, *Veillonella* and *Leptotrichia*. However, these microbiota profiles were observed in both in mid-aged adults and elderly. The aim of this study was to determine whether nasal and/or oropharyngeal microbiota profiles are associated with age and RTIs.

## Methods

### Source of samples

Between Augustus 2012 and Augustus 2014, respiratory swabs were collected from adult patients who were visiting the otorhinolaryngology outpatient clinic or hospitalized at the pulmonary ward of the Reiner de Graaf Gasthuis (Delft, The Netherlands). Two swabs were collected from each patient using sterile flocked swabs (Puritan Medical Products, Maine, USA). One swab was obtained from the head of the concha inferior near the anterior nares and a second swab was obtained from the oropharynx. Each swab was stored in 2 mL STGG (skim milk, tryptone, glucose, glycerol) medium. In total, swabs of 370 patients without clinical symptoms of an RTI and of 211 patients with a suspected URTI or LRTI were collected. For the current analysis, swabs collected from patients with a suspected RTI who received antibiotics in the week before visiting the outpatient clinic and swabs collected from hospitalized patients > 1 day after admission were excluded (*n* = 59 patients), leaving swabs of 152 patients with an RTI for further analysis. Subsequently, swabs of 152 patients without clinical symptoms (i.e. controls) were selected based on sample collection date, age and sex to match the patient group so well as possible. None of them had received antibiotics 1 week prior to sample collection.

### Nucleic acid extraction and sequencing

Nucleic acids were extracted from 500 μL STGG medium and eluted in a final volume of 100 μL with the MagNA Pure 96 instrument using the MagNA Pure 96 DNA and Viral NA Large Volume kit and the Pathogen Universal protocol (Roche Diagnostics, Basel, Switzerland). Amplicon sequencing of the 16S ribosomal RNA (rRNA) gene was performed as described elsewhere [20]. Briefly, a fragment of ~ 464 bp of the V3-V4 region of the 16S rRNA gene was amplified and sequenced with the MiSeq desktop sequencer (Illumina, San Diego, USA).

### Microbiota analysis

Sequencing data was processed following the QIIME1 pipeline. Open reference OTU clustering of high-quality sequences (≥ 100 bp in length with a quality score ≥ Q20) was conducted using UCLUST at a 97% similarity level against a pre-clustered version of the Augustus 2013 GreenGenes database. No low abundance filtering was used. See for further details Additional file 1. Operational taxonomic units (OTUs) with *Alloiococcus* or *Propionibacterium* annotation were renamed. *Dolosigranulum* is known to be misclassified in the GreenGenes database as *Alloiococcus* [21]. BLAST search confirmed that the representative sequence matched *Dolosigranulum* in BLAST. *Propionibacterium* spp. have been reclassified to the genus *Cutibacterium* [22].

### *Moraxella* species determination

*Moraxella* species were identified using quantitative real-time PCR (qPCR) and culture. A highly specific qPCR targeting the *copB* gene of *Moraxella catarrhalis* was performed on the isolated DNA of all samples and performed as described elsewhere [20]. Culture was performed to determine which *Moraxella* spp. was present in the samples negative for *M. catarrhalis*. For culture, 200–300 μL STGG medium was inoculated on blood agar plates (Becton, Dickinson and Company, New Jersey, USA) and incubated at 35 °C in a 5% CO2 incubator. Species were identified by matrix-assisted laser desorption ionization time of flight mass spectrometry (MALDI-TOF MS) analyzer with software version 1.6.7.1000 (Bruker corporation, Billerica, USA).

### Statistical analysis

For statistical analysis the software package SPSS version 26 was used. Statistically significant differences in variables between the controls and the patients with an RTI was calculated using the Mann-Whitney U and chi-square test for continuous and categorical data, respectively. After the core members of the nasal and oropharyngeal microbiota were determined, hierarchical clusters of microbiota profiles were defined using the free python script ‘hierarchical_clustering.py’, which was written by Nathan Salomonis of the J. David Gladstone Institutes (San Francisco, CA, USA) and can be found on the following webpage: https://github.com/nsalomonis/altanalyze/blob/master/visualization_scripts/clustering.py. This script uses the Euclidean distance to measure the dissimilarity between each pair of observations. The prevalence of each microbiota cluster per age group was calculated for the controls and the patients with an RTI. Subsequently, the Fisher’s Exact test was performed to determine whether microbiota clusters were associated with age and/or RTIs. For the cluster associated with age and RTIs, Fisher’s Exact tests were performed to determine whether season of sampling, sex, smoking, young children at home, comorbidities, the use of inhaler or nasal spray were also associated with this cluster in the control group. Furthermore, statistically significant differences in the relative abundance of the genus *Moraxella* between groups was calculated using the Mann-Whitney U test.

## Results

### Study population

Nasal and oropharyngeal swabs of 152 controls and 152 patients with an RTI were selected (Table 1). The 152 controls were visiting the outpatient clinic mainly for an audiogram or hearing complaints (37%), or allergy, skin test or immunotherapy (24%). Of the 152 patients with an RTI, 72 (47%) were suffering from an URTI (i.e. a common cold, sinusitis, tonsillitis or laryngitis). The remaining 80 (53%) patients were hospitalized with a LRTI (i.e. a pneumonia, chronic obstructive pulmonary disease exacerbation, bronchitis or asthma exacerbation), which was diagnosed by the treating physician. Both groups differed significantly in age (*P* = 0.013).

**Table 1:** Population characteristics

Group	Controls (*n* = 152)	Patients with a respiratory tract infection (*n* = 152)
Age, mean ± SD (range)*	53 ± 19 (18–92)	58 ± 20 (18–89)
Age category, n (%)**		
< 65 years	102 (67)	81 (53)
≥ 65 years	50 (33)	71 (47)
Sex, n (%)		
Female	79 (52)	86 (57)
Male	73 (48)	66 (43)
Season of sampling, n (%)		
Autumn	40 (26)	33 (22)
Winter	47 (31)	59 (39)
Spring	35 (23)	44 (29)
Summer	30 (20)	16 (11)
Reason for visit/hospitalisation, n (%)		
Allergy/skin test/immunotherapy	37 (24)	5 (3)
Audiogram/hearing complaints	56 (37)	7 (5)
Dizzines	9 (6)	0 (0)
Infection	0 (0)	110 (72)
Follow-up	10 (7)	7 (5)
Nose spray	0 (0)	6 (4)
Other; ears^a^	23 (15)	7 (5)
Other; nose^b^	10 (7)	4 (3)
Other; throat^c^	7 (5)	2 (1)
Other; accompaniment	0 (0)	4 (3)
Upper airway infection, n (%)		
Common cold	Not applicable	47 (31)
Laryngitis		4 (3)
Sinusitis		14 (9)
Tonsillitis		7 (5)
Lower airway infection, n (%)		
Asthma exacerbation	Not applicable	2 (1)
Bronchitis		3 (2)
COPD exacerbation		24 (16)
Pneumonia		51 (34)

*Abbreviations*: *COPD* Chronic obstructive pulmonary disease, *SD* Standard deviation. Statistically significant differences in variables between both groups was calculated using the Mann-Whitney U and chi-square test for continuous and categorical data, respectively. *P = 0.013. **P = 0.014

^a^Other; ears included cleaning of ears and inserting grommets

^b^Other; nose included septum deviation, frequent nosebleeds and choanal polyp

^c^Other; throat included complaints of long-lasting cough or difficult swallowing movement

### Determination of the core microbiota of the nasal passages and oropharynx

To determine whether the nasal and/or oropharyngeal microbiota were associated with age and RTIs, first core microbiota profiles were defined using amplicon sequencing of the 16S rRNA gene. A mean of 77,414 reads per swab were obtained with sequencing, which resulted in a mean OTU of 50 for the nasal swabs and 83 for the oropharyngeal swabs.

In the nasal passages of the 152 controls and 152 patients with an RTI, the 10 most abundant genera/families were *Corynebacterium* (mean relative abundance of 28%), *Staphylococcus* (24%), *Moraxella* (12%), *Dolosigranulum* (7%), *Streptococcus* (5%), *Haemophilus* (3%), *Peptoniphilus* (3%), *Cutibacterium* (2%), *Anaerococcu*s (2%), and Enterobacteriaceae (2%). Together these bacteria account for 88% of the classified sequences. Interestingly, three microbiota profiles were dominated (i.e. ≥ 50% relative abundance) by one of the less abundant genera *Pseudomonas* and *Neisseria.*

In the oropharynx, *Prevotella* (mean relative abundance of 26%), *Veillonella* (16%), *Streptococcus* (11%), *Neisseria* (7%), *Fusobacterium* (6%), *Leptotrichia* (5%), *Haemophilus* (5%), *Rothia* (3%), *Porphyromonas* (3%), and *Actinobacillus* (2%) were the 10 most abundant genera, accounting for 84% of the classified sequences. Four microbiota profiles were dominated by *Lactobacillus* or *Staphylococcus*.

These bacteria are the core members of the nasal and oropharyngeal microbiota of the controls and patients with an RTI. Separate analyses for both patient groups resulted in comparable core members (Additional file 2).

### Microbiota clustering analysis based on nasal or oropharyngeal core members

To define clusters of microbiota profiles, hierarchical clustering was performed based on the nasal or oropharyngeal core members (Additional file 3a, b). For the nasal passages, eight microbiota clusters were defined (Additional file 4a). Cluster I was characterized by a relatively high abundance of *Haemophilus*, *Neisseria* or *Streptococcus* (Hae/Nei/Str), II by *Moraxella* (Mor), III by *Staphylococcus* and *Corynebacterium* (Sta, Cor), IV by *Corynebacterium* and *Dolosigranulum* (Cor, Dol), V by *Corynebacterium* (Cor), VI by *Staphylococcus* (Sta), VII by *Moraxella* and *Corynebacterium* (Mor, Cor), and VIII by *Dolosigranulum*, *Haemophilus*, *Cutibacterium*, Enterobacteriaceae or *Streptococcus* (Dol/Hae/Cut/Ent/Str). These microbiota clusters had a mean Shannon diversity index ranging between 2.18 and 4.50.

For the oropharynx, nine microbiota clusters were defined of which cluster I was characterized by a relatively high abundance of *Prevotella* and *Fusobacterium* (Pre, Fus), II/III by *Prevotella* and *Veillonella* (Pre, Vei), IV by *Prevotella* (Pre), V by *Actinobacillus*, *Haemophilus*, *Staphylococcus*, *Rothia* or *Neisseria* (Act/Hae/Sta/Rot/Nei), VI by *Streptococcus* and *Veillonella* (Str, Vei), VII by *Lactobacillus* (Lac), VIII by *Streptococcus* and *Rothia* (Str, Rot), and IX by *Streptococcus*, *Neisseria*, *Actinobacillus*, *Lactobacillus* or *Staphylococcus* (Str/Nei/Act/Lac/Sta) (Additional file 4b). Compared to the nasal microbiota clusters, the microbiota profiles within the oropharyngeal microbiota clusters were more variable which was illustrated by the mean Shannon diversity index ranging between 3.13 and 6.45. There was no correlation between the nasal and oropharyngeal microbiota clusters.

### Nasal and oropharyngeal microbiota clusters related to higher age in the control group

**Fig. 1:**
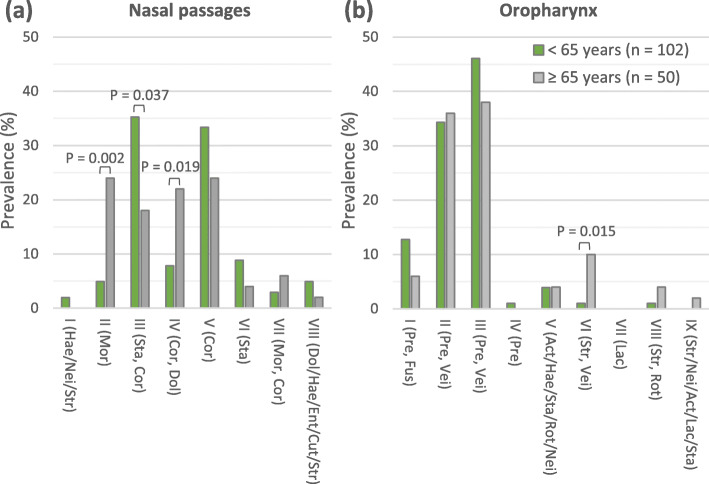
Prevalence of microbiota clusters among the controls aged < 65 and ≥ 65 years. **a** nasal microbiota clusters. **b** oropharyngeal microbiota clusters. Act: *Actinobacillus*; Cor: *Corynebacterium*; Cut: *Cutibacterium*; Dol: *Dolosigranulum*; Ent: Enterobacteriaceae; Fus: *Fusobacterium*; Hae: *Haemophilus*; Lac: *Lactobacillus*; Mor: *Moraxella*; Nei: *Neisseria*; Pre: *Prevotella*; Rot: *Rothia*; Sta: *Staphylococcus;* Str: *Streptococcus*; Vei: *Veillonella*. Genera separated from each other by a comma are both represented in a relatively high abundance in each microbiota profile of the relevant cluster. Genera separated from each other by a slash indicates that one of these genera is present in a relatively high abundance. All *p*-values are based on Fisher’s Exact test. Correction for multiple testing was not performed

After clusters of nasal and oropharyngeal microbiota profiles were defined, their prevalence in the control group was calculated per age group (adults aged ≥65 years or < 65 years). Of the eight nasal microbiota clusters, three microbiota clusters were associated with age (Fig. 1a). Cluster II (Mor) and IV (Cor, Dol) were significantly more prevalent in adults aged ≥65 years compared to adults aged < 65 years (*P* ≤ 0.019), whereas cluster III (Sta, Cor) was significantly less prevalent in adults aged ≥65 years (*P* = 0.037). Of the nine oropharyngeal microbiota clusters, cluster VI (Str, Vei) was significantly more prevalent in adults aged ≥65 years (*P* = 0.015; Fig. 1b). These data showed that specific microbiota profiles of both the nasal passages and oropharynx are associated with higher age.

### Nasal and oropharyngeal microbiota clusters related to higher age and RTIs

**Fig. 2:**
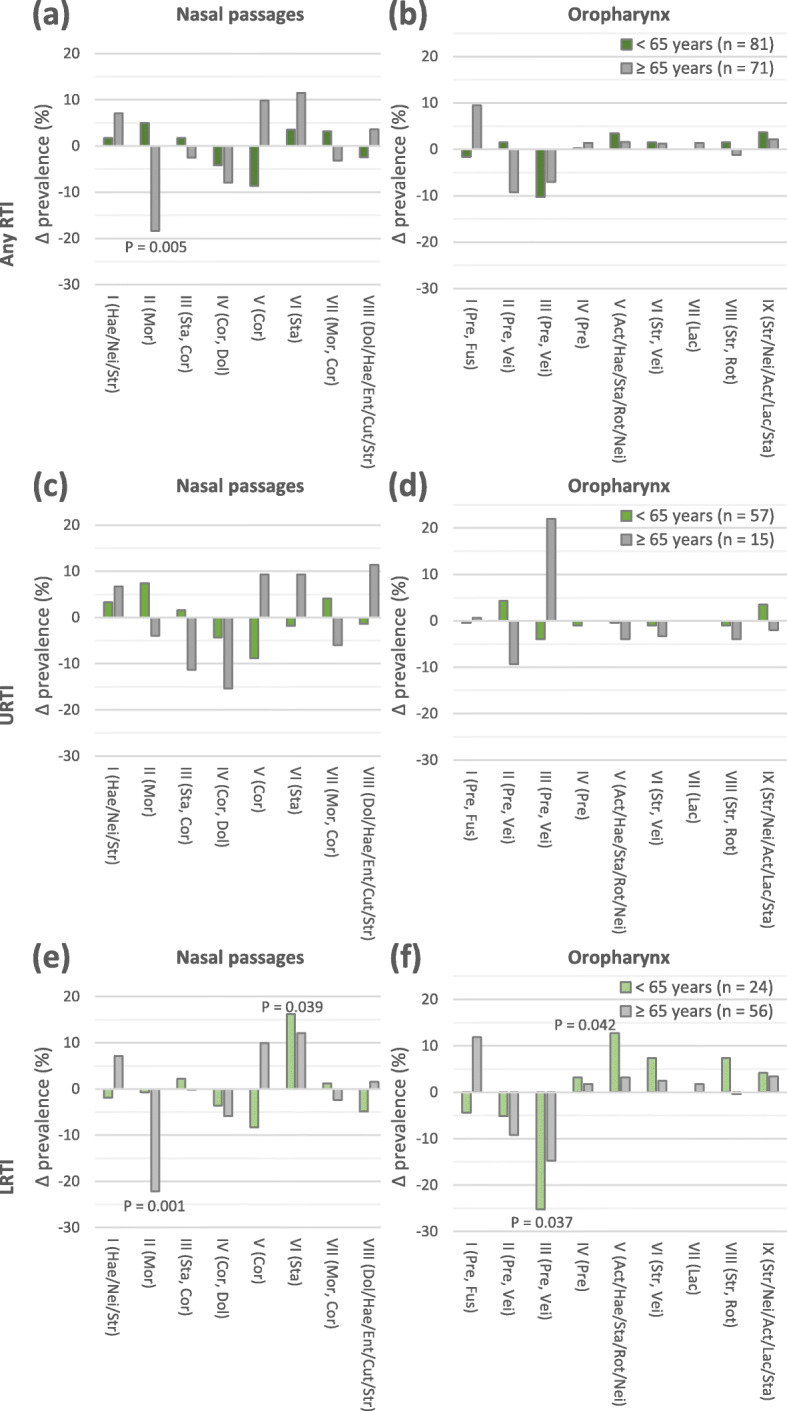
Comparison of prevalence of microbiota clusters between controls and patients per age group. **a** nasal and **b** oropharyngeal microbiota clusters of controls and patients with any respiratory tract infection (RTI). **c** nasal and **d** oropharyngeal microbiota clusters of controls and patients with an upper respiratory tract infection (URTI). **e** nasal and **f** oropharyngeal microbiota clusters of controls and patients with a lower respiratory tract infection (LRTI). Act: *Actinobacillus*; Cor: *Corynebacterium*; Cut: *Cutibacterium*; Dol: *Dolosigranulum*; Ent: Enterobacteriaceae; Fus: *Fusobacterium*; Hae: *Haemophilus*; Lac: *Lactobacillus*; Mor: *Moraxella*; Nei: *Neisseria*; Pre: *Prevotella*; Rot: *Rothia*; Sta: *Staphylococcus;* Str: *Streptococcus*; Vei: *Veillonella*. Genera separated from each other by a comma are both represented in a relatively high abundance in each microbiota profile of the relevant cluster. Genera separated from each other by a slash indicates that one of these genera is present in a relatively high abundance. All *P*-values are based on Fisher’s Exact test. Correction for multiple testing was not performed

Subsequently, the prevalence of the nasal and oropharyngeal microbiota clusters of the patients with any RTI (Fig. 2a, b), URTI (Fig. 2c, d) or LRTI (Fig. 2e, f) were compared to the control group. Nasal microbiota cluster II (Mor) was strongly associated with higher age and LRTI as it was significantly less prevalent in patients with a LRTI who passed the age of 65 years compared to controls with similar age (*P* = 0.001).

Nasal microbiota cluster VI (Sta; *P* = 0.039), oropharyngeal microbiota cluster III (Pre, Vei; *P* = 0.037) and oropharyngeal microbiota cluster V (Act/Hae/Sta/Rot/Nei; *P* = 0.042) were moderately associated with LRTIs in patients aged < 65 years. Interestingly, oropharyngeal microbiota cluster VII (Lac) was only present in patients with a LRTI who passed the age of 65 years. These data indicate that both the nasal and oropharyngeal microbiota differed between the controls and patients with a LRTIs.

### Nasal microbiota cluster II dominated by *Moraxella* spp.

Of all identified microbiota clusters, nasal cluster II (Mor) was of most interest since it was associated with higher age and less prevalent in elderly with a LRTI compared to the healthy elderly population. This finding was strengthened by the significant difference in mean abundance of *Moraxella* spp. between the age groups of the controls (*P* = 0.003) and between the controls and patients with a LRTI who passed the age of 65 years (*P* = 0.008; Table 2). In the control group, no association with season of sampling, sex, smoking, young children at home, comorbidities, the use of inhaler or nasal spray was found.

**Table 2:** Mean relative abundance of *Moraxella* spp. per population group

Population group	All ages		< 65 years		≥ 65 years	
	Mean ± SD (%)	Range (%)	Mean ± SD (%)	Range (%)	Mean ± SD (%)	Range (%)
Controls and patients	12 ± 26	0–100	9 ± 23	0–100	15 ± 29	0–100
Controls	13 ± 28	0–100	7 ± 20*	0–99	25 ± 37 */**/***	0–100
Patients	10 ± 24	0–100	12 ± 27	0–100	8 ± 20**	0–81
Patients with URTI	15 ± 29	0–100	14 ± 29	0–100	15 ± 30	0–81
Patients with LRTI	6 ± 18	0–100	8 ± 23	0–100	6 ± 15***	0–67

*Abbreviations*: *LRTI* Lower respiratory tract infection, *SD* Standard deviation, *URTI* Upper respiratory tract infection. Statistically significant differences between groups was calculated using the Mann-Whitney U test. *P = 0.003. **P = 0.018. ***P = 0.008

To determine whether *M. catarrhalis* was representing nasal cluster II (Mor), a qPCR was performed. Of all 29 nasal swabs, five (18%) were positive for *M. catarrhalis*. Culture data suggested that the remaining 24 (82%) of the swabs within nasal cluster II (Mor) were represented by *M. nonliquefaciens*.

## Discussion

To the best of our knowledge, this is the largest study on the nasal and oropharyngeal microbiota and its relation to both URTIs and LRTIs in elderly. Based on the microbiota profiles of the controls and the patients with an RTI, we defined eight nasal and nine oropharyngeal microbiota clusters. One of the nasal microbiota clusters was strongly associated with age and RTIs.

The results of this study showed that nasal cluster II dominated by *M. catarrhalis/nonliquefaciens*, was significantly more prevalent in the healthy elderly population compared to the healthy mid-aged adults. Interestingly, *M. catarrhalis/nonliquefaciens* was significantly less prevalent in elderly with a LRTI compared to the healthy elderly population, suggesting an association between *M. catarrhalis/nonliquefaciens* and respiratory health in elderly. Previous reports have shown that *Moraxella* spp. become predominant community members over time in most young children [5–9]. Their microbiota as well as their immune system are in development, whereas both innate and adaptive immunity seem to deteriorate in elderly [11]. In essence, it might tolerate the same bacterial species. This might explain the significantly higher prevalence of *M. catarrhalis/nonliquefaciens* in the healthy elderly population. However, conflicting results have been reported regarding the role of *Moraxella* spp. in the pathogenesis of RTIs in young children. Some studies found that profiles dominated by *M. catarrhalis/nonliquefaciens* was associated with respiratory health [5–8], while others reported that *Moraxella* spp. were associated with high susceptibility to LRTIs [9]. Since *M. catarrhalis* has been considered as being a pathogen for certain disease entities (e.g. COPD exacerbation and otitis media), it is most likely that *M. nonliquefaciens* is actually associated with respiratory health.

Nasal and oropharyngeal microbiota clusters moderately associated with LRTIs were characterized by a relatively high abundance of a potential pathogen, such as *Staphylococcus*, *Actinobacillus*, *Haemophilus*, and *Rothia* spp. The difference in prevalence compared to the healthy population was observed in both age groups but was only significantly different in the mid-age adults. This means that no microbiota cluster was defined that could elucidate why elderly are more susceptibility to LRTIs. However, the data does indicate that both the nasal and oropharyngeal microbiota have impact on lower airway health in adults while it is generally assumed that only the oropharynx is involved in the pathogenesis of LRTI [22].

A cross-sectional study of Steenhuijsen Piters and colleagues revealed 11 (sub)clusters of oropharyngeal microbiota profiles [18]. Three clusters were associated with pneumonia which were characterized by a relative high abundance of *S. (pseudo)pneumoniae*, *Rothia* spp. or *Lactobacillus* spp. In contrast, three other microbiota clusters were correlated with respiratory health and contained high abundances of *Prevotella*, *Veillonella* and *Leptotrichia*. In our study, *Streptococcus*, *Rothia* and *Lactobacillus* dominated only a limited number of oropharyngeal microbiota profiles. Notably, the oropharyngeal microbiota cluster characterized by a relatively high abundance of *Lactobacillus* was only covered by patients with a LRTI. Furthermore, we observed a moderate association between an oropharyngeal microbiota cluster with high abundances of *Prevotella* and *Veillonella* and respiratory health in mid-aged adults.

URTIs are mainly caused by viruses and previous reports have shown that *Streptococcus* and *Haemophilus* spp. are associated with viral infections [23–26]. The interactions between viruses and the airway microbiota may affect the course of the disease and subsequent respiratory health [27]. In our study, nasal microbiota clusters characterized by a high abundance of *Streptococcus* or *Haemophilus* spp. were associated with the presence of respiratory viruses in patients with a URTI (data not shown). However, no significant difference in prevalence was observed compared to the healthy population.

A limitation of this study is that the data was collected at one timepoint. Longitudinal and more comprehensive data regarding microbiota composition and function as well as immunogenic status is required in order to elucidate the mechanism of the heightened susceptibility to RTIs in elderly. Longitudinal data is also required to confirm that nasal microbiota has impact on the lower airway health in adults. Stronger correlations might have been found when data was used from a matched case-control study, controls were healthy relatives of the patients, only patients with a confirmed pneumonia were included, nasopharynx was sampled and when specimens were collected during hospital admission, reducing antibiotic usage prior to specimen collection. Lastly, sputum collection in case of a LRTI would have been valuable to identify the causative pathogen [20].

## Conclusions

We showed that nasal microbiota dominated by *M. catarrhalis/nonliquefaciens* is associated with respiratory health in the elderly population. Further research is required to determine which species is associated with respiratory health and whether it is a positive association. In case of a positive association, efforts should be made to uphold these bacteria to promote respiratory health in the elderly population.

## Supplementary information

**Additional file 1.** Qiime1 version 1.9.1 Scripts and settings.

**Additional file 2 **Core microbiota members of the (**a**) nasal passages or (**b**) oropharynx based on the profiles of controls and/or patients with a respiratory tract infection (RTI).

**Additional file 3 **Hierarchical clustering of (**a**) the nasal and (**b**) the oropharyngeal microbiota profiles of the 152 controls and 152 patients with a respiratory tract infection based on the core members.

**Additional file 4 **Characteristics of (**a**) nasal and (**b**) oropharyngeal microbiota clusters based on the core members of the 152 controls and 152 patients with a respiratory tract infection.

## Data Availability

The datasets generated datasets are available in the NCBI Sequence Read Archive (https://www.ncbi.nlm.nih.gov/sra) repository with the accession number PRJNA596902.

## Abbreviations

Act: Actinobacillus
COPD: Chronic obstructive pulmonary disease
Cor: Corynebacterium
Cut: Cutibacterium
Dol: Dolosigranulum
Ent: Enterobacteriaceae
Fus: Fusobacterium
Hae: Haemophilus
Lac: Lactobacillus
LRTIs: Lower respiratory tract infections
Nei: Neisseria
MALDI-TOF MS: Matrix-assisted laser desorption ionization time of flight mass spectrometry
Mor: Moraxella
OTUs: Operational taxonomic units
Pre: Prevotella
qPCR: Quantitative real-time PCR
Rot: Rothia
rRNA: Ribosomal RNA
RTIs: Respiratory tract infections
Sta: Staphylococcus
STGG: Skim milk, tryptone, glucose, glycerol
Str: Streptococcus
URTIs: Upper respiratory tract infections
Vei: Veillonella
