# Supplementary material for: Nasal microbiota dominated by Moraxella spp. is associated with respiratory health in the elderly population: a case control study
Source: Respir Res. 2020 Jul 14;21:181. doi: 10.1186/s12931-020-01443-8 (PMC7362441; doi:10.1186/s12931-020-01443-8)
Supplement: Supplementary file 1 — Additional file 1. Qiime1 version 1.9.1 Scripts and settings. [file 12931_2020_1443_MOESM1_ESM.pdf]

**Scripts used (per sample)**	**Extra settings from default**	**Description**
Trimmomatic v0.38 (non-Qiime script)	-threads 12 MINLEN:100	Read length threshold filter
join_paired_ends	--perc_max_diff 15	Merge paired-end NGS data
split_libraries_fastq	-q 19 --barcode_type 'not-barcoded'	Quality filter
pick_open_reference_otus	<default settings>	OTU picking (classification)
biom summarize-table	<default settings>	Summarize biom table
Filter on sample depth based on biom summary (non-Qiime script, custom python script)	Read depth threshold: 5000 reads	Sample depth filtering on mapping file
filter_samples_from_otu_table	--sample_id_fp <filtered mapping file>	Sample filtering on biom file
core_diversity_analyses	-e 8369 -a -O 20 -p <parameters file> for nose dataset<br>-e 6881 -a -O 20 -p <parameters file> for throat dataset<br><br>Core diversity parameters:<br>- alpha_diversity:metrics observed_species,chao1,shannon,PD_whole_tree<br>- beta_diversity:metrics bray_curtis,euclidean,unweighted_unifrac,weighted_unifrac	Alpha & Beta diversity analysis
