# Supplementary material for: Nasal microbiota dominated by Moraxella spp. is associated with respiratory health in the elderly population: a case control study
Source: Respir Res. 2020 Jul 14;21:181. doi: 10.1186/s12931-020-01443-8 (PMC7362441; doi:10.1186/s12931-020-01443-8)
Supplement: Supplementary file 2 — Additional file 2 Core microbiota members of the (a) nasal passages or (b) oropharynx based on the profiles of controls and/or patients with a respiratory tract infection (RTI). [file 12931_2020_1443_MOESM2_ESM.pdf]

(**a**) Nasal passages

Controls and patients with an RTI (n = 304)		Controls (n = 152)		Patients with an RTI (n = 152)	
Top 10 most abundant genera/families (fraction of total reads)					
1	*Corynebacterium* (28%)	1	*Corynebacterium* (29%)	1	*Corynebacterium* (25%)
2	*Staphylococcus* (24%)	2	*Staphylococcus* (22%)	2	*Staphylococcus* (25%)
3	*Moraxella* (12%)	3	*Moraxella* (13%)	3	*Moraxella* (10%)
4	*Dolosigranulum* (7%)	4	*Dolosigranulum* (8%)	4	*Streptococcus* (7%)
5	*Streptococcus* (5%)	5	*Peptoniphilus* (3%)	5	*Dolosigranulum* (7%)
6	*Haemophilus* (3%)	6	*Cutibacterium* (3%)	6	*Haemophilus* (5%)
7	*Peptoniphilus* (3%)	7	*Streptococcus* (3%)	7	*Peptoniphilus* (3%)
8	*Cutibacterium* (2%)	8	*Anaerococcus* (2%)	8	Enterobacteriaceae (2%)
9	*Anaerococcus* (2%)	9	*Neisseria* (2%)	9	*Pseudomonas* (2%)
10	*Enterobacteriaceae* (2%)	10	*Haemophilus* (1%)	10	*Prevotella* (2%)
Other genera with ≥ 50% relative abundance in at least one swab (number of swabs)					
12	*Pseudomonas* (1)	11	Enterobacteriaceae (1)		
14	*Neisseria* (2)				

(**b**) Oropharynx

Controls and patients with an RTI (n = 304)		Controls (n = 152)		Patients with an RTI (n = 152)	
Top 10 most abundant genera/families (fraction of total reads)					
1	*Prevotella* (26%)	1	*Prevotella* (27%)	1	*Prevotella* (24%)
2	*Veillonella* (16%)	2	*Veillonella* (16%)	2	*Veillonella* (16%)
3	*Streptococcus* (11%)	3	*Streptococcus* (10%)	3	*Streptococcus* (12%)
4	*Neisseria* (7%)	4	*Fusobacterium* (7%)	4	*Neisseria* (7%)
5	*Fusobacterium* (6%)	5	*Neisseria* (6%)	5	*Leptotrichia* (5%)
6	*Leptotrichia* (5%)	6	*Leptotrichia* (6%)	6	*Haemophilus* (4%)
7	*Haemophilus* (5%)	7	*Haemophilus* (5%)	7	*Fusobacterium* (4%)
8	*Rothia* (3%)	8	*Porphyromonas* (3%)	8	*Rothia* (3%)
9	*Porphyromonas* (3%)	9	*Actinobacillus* (2%)	9	*Lactobacillus* (3%)
10	*Actinobacillus* (2%)	10	*Rothia* (2%)	10	*Actinobacillus* (2%)
Other genera with ≥ 50% relative abundance in at least one swab (number of swabs)					
13	*Lactobacillus* (3)			16	*Staphylococcus* (1)
21	*Staphylococcus* (1)				
