# Supplementary figures and images for: Nasal microbiota dominated by Moraxella spp. is associated with respiratory health in the elderly population: a case control study
Source: Respir Res. 2020 Jul 14;21:181. doi: 10.1186/s12931-020-01443-8 (PMC7362441; doi:10.1186/s12931-020-01443-8)

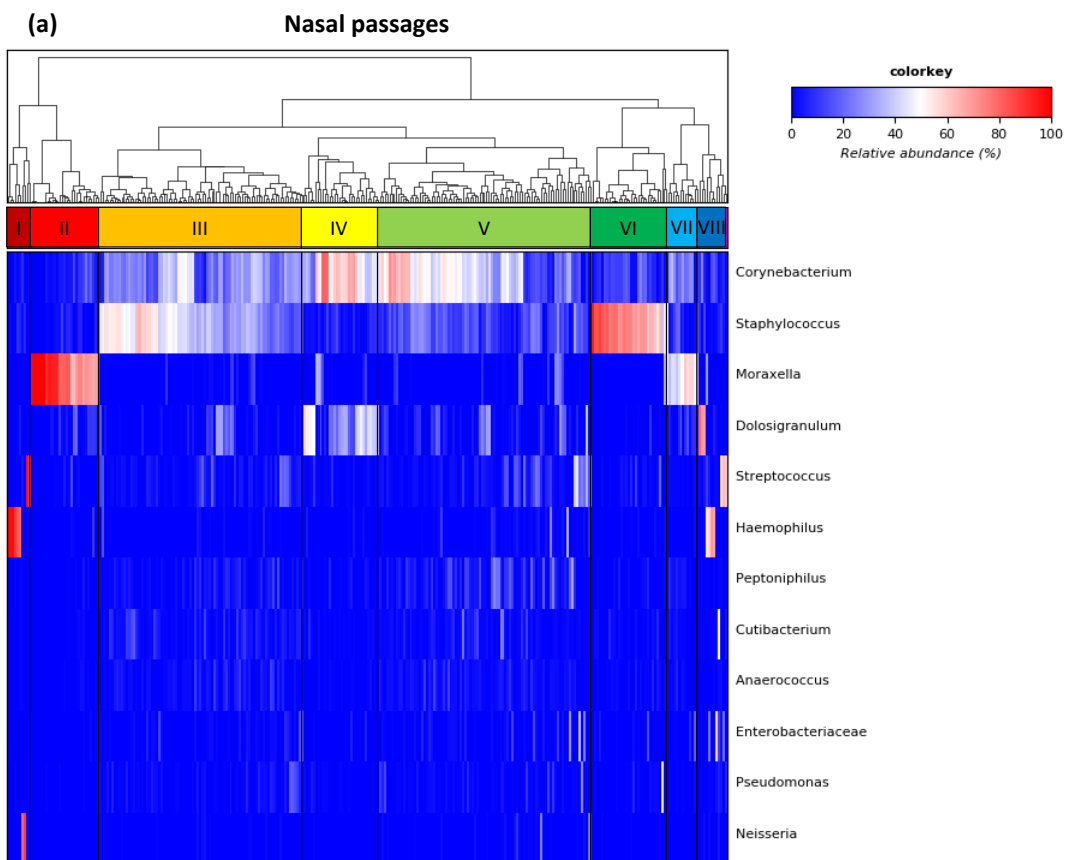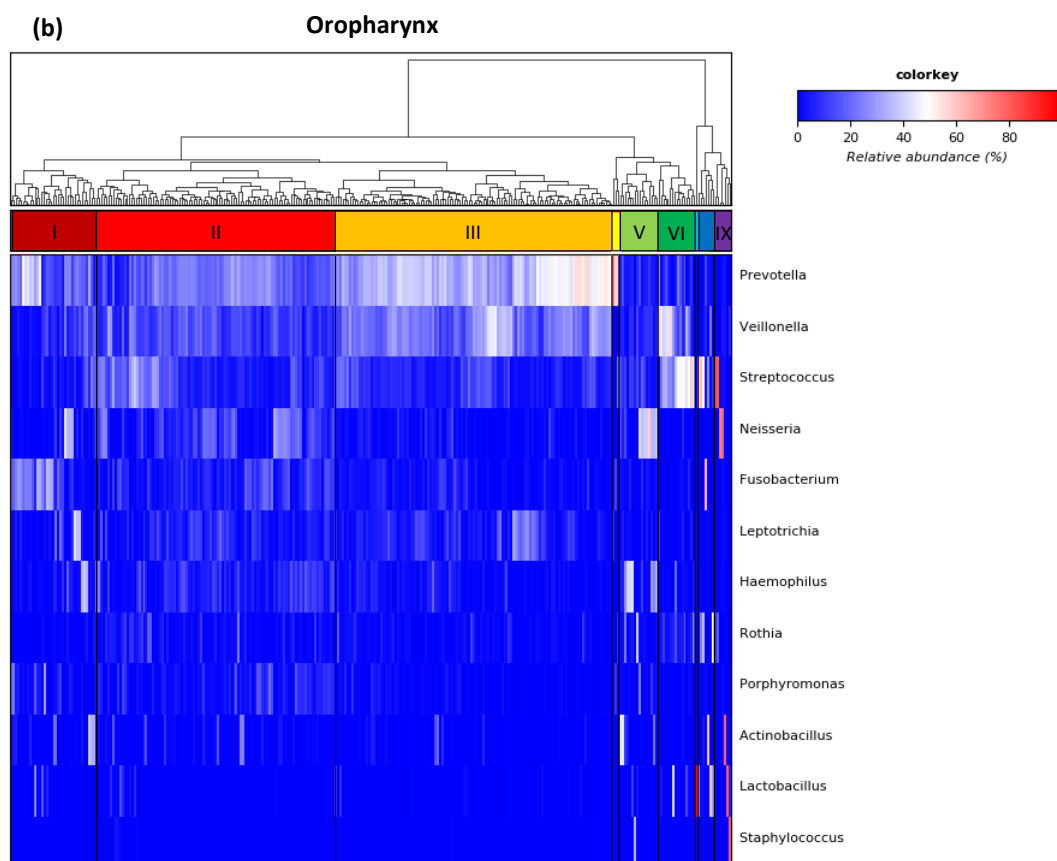

Supplement: Supplementary file 3 — Additional file 3 Hierarchical clustering of (a) the nasal and (b) the oropharyngeal microbiota profiles of the 152 controls and 152 patients with a respiratory tract infection based on the core members. [file 12931_2020_1443_MOESM3_ESM.pdf]
