# Supplementary material for: Nasal microbiota dominated by Moraxella spp. is associated with respiratory health in the elderly population: a case control study
Source: Respir Res. 2020 Jul 14;21:181. doi: 10.1186/s12931-020-01443-8 (PMC7362441; doi:10.1186/s12931-020-01443-8)
Supplement: Supplementary file 4 — Additional file 4 Characteristics of (a) nasal and (b) oropharyngeal microbiota clusters based on the core members of the 152 controls and 152 patients with a respiratory tract infection. [file 12931_2020_1443_MOESM4_ESM.pdf]

(a) Nasal passages

Cluster	Number of swabs (%)	Genera (relative abundance range)	Shannon diversity
I (Hae/Nei/Str)	10 (3)	*Haemophilus* (79-100), *Neisseria* (78-87) or *Streptococcus* (86-97)	2.18 ± 1.07
II (Mor)	29 (10)	*Moraxella* (62-100)	2.95 ± 0.99
III (Sta, Cor)	86 (28)	*Staphylococcus* (11-65) and *Corynebacterium* (3-53)	4.45 ± 0.81
IV (Cor, Dol)	32 (11)	*Corynebacterium* (19-81) and *Dolosigranulum* (0-58)	3.65 ± 0.90
V (Cor)	90 (30)	*Corynebacterium* (0-73)	4.50 ± 0.69
VI (Sta)	32 (11)	*Staphylococcus* (37-95)	3.74 ± 0.77
VII (Mor, Cor)	13 (4)	*Moraxella* (36-61) and *Corynebacterium* (1-37)	4.19 ± 0.60
VIII (Dol/Hae/Ent/Cut/Str)	12 (4)	*Dolosigranulum* (69-80), *Haemophilus* (54-70), Enterobacteriaceae (57), *Cutibacterium* (51) or *Streptococcus* (57-63)	3.76 ± 0.78

(b) Oropharynx

Cluster	Number of swabs (%)	Genera (relative abundance range)	Shannon diversity
I (Pre, Fus)	36 (12)	*Prevotella* (4-48) and *Fusobacterium* (4-36), *Neisseria* (34-45), *Leptotrichia* (36-44), *Haemophilus* (35-39) or *Actinobacillus* (33-40)	5.64 ± 0.88
II (Pre, Vei)	101 (33)	*Prevotella* (2-34) and *Veillonella* (1-32)	6.45 ± 0.74
III (Pre, Vei)	117 (38)	*Prevotella* (18-57) and *Veillonella* (7-47)	6.06 ± 0.82
IV (Pre)	3 (1)	*Prevotella* (55-75)	4.61 ± 1.51
V (Act/Hae/Sta/Rot/Nei)	16 (5)	*Actinobacillus* (10-45), *Haemophilus* (41-45), *Staphylococcus* (35), *Rothia* (41) or Neisseria (35-58)	4.87 ± 0.51
VI (Str, Vei)	16 (5)	*Streptococcus* (21-59) and *Veillonella* (1-58)	4.81 ± 0.48
VII (Lac)	1 (0)	*Lactobacillus* (98)	5.03
VIII (Str, Rot)	7 (2)	*Streptococcus* (1-61) and *Rothia* (0-49)	4.05 ± 0.79
IX (Str/Nei/Act/Lac/Sta)	7 (2)	*Streptococcus* (79-86), *Neisseria* (74-77), *Actinobacillus* (80), *Lactobacillus* (80) or *Staphylococcus* (79)	3.13 ± 0.47
